# The effect of juggling on the proprioceptive and attentional abilities among older women

**DOI:** 10.3389/fpubh.2024.1386981

**Published:** 2024-10-02

**Authors:** Jakub Malik, Natalia Główka, Wojciech Jelonek, Janusz Maciaszek

**Affiliations:** ^1^Department of Physical Activity and Health Promotion Science, Poznan University of Physical Education, Poznan, Poland; ^2^Department of Sports Dietetics, Poznan University of Physical Education, Poznan, Poland; ^3^Department of Neuromuscular Physiotherapy, Poznan University of Physical Education, Poznan, Poland

**Keywords:** attention, bilateral exercises, dual-task, joint position matching, physical activity, reaction time

## Abstract

**Background:**

Age-related changes in attentional abilities can lead to a decline in body segment awareness in space. However, studies have reported that physical activity can improve proprioception among older adults, although proven activities with this potential are limited. Juggling is a promising activity for enhancing proprioception, as it requires high levels of attention and sensory precision. The first hypothesis posited that a juggling intervention would positively impact ipsilateral and contralateral elbow joint position matching without visual input. The second hypothesis suggested a correlation between cognitive abilities and joint position sense efficiency.

**Methods:**

A total of 20 older women (mean age: 69.95 ± 4.58) participated in a repeated-measures study using a Latin square design. Measurements were taken at three time points (baseline, post-juggling, and control). Ipsilateral and contralateral elbow joint position matchings without visual or verbal feedback of accuracy were used to assess proprioception. Attention and reaction time variables were measured using the Vienna Test System protocols.

**Results:**

Although significant changes were observed between baseline and subsequent time points in joint position sense accuracy, no specific effect of juggling was detected. Low and medium correlations were found between decision time and the variability of choice reaction time with contralateral accuracy. For ipsilateral accuracy, a relationship was observed only with handedness. No correlations were found between attention test scores and joint position sense accuracy.

**Conclusion:**

The study did not demonstrate a significant effect of juggling on position-matching ability. However, cognitive abilities such as decision speed and the stability of choice reaction time may play a role in enhancing position-matching in older women.

**Clinical trial registration:**

ClinicalTrials.gov, identifier NCT06108713.

## 1 Introduction

The level of physical fitness decreases with age, which can impair functional performance, particularly in activities of daily living (1, 2). A loss of coordination, often contributing to an increased risk of falls, is a key factor in functional deterioration and may be associated with decreased proprioception (2). By the end of 2020, approximately 25% of Poland’s population was aged ≥60 years, with women comprising the majority (51). Although women tend to live longer than men, they are also more likely to experience chronic diseases and disabilities (3). Additionally, the age-related decline in physical activity is more pronounced in women than in men (4), which may contribute to a less active lifestyle among older women (5).

Proprioception plays an important role in age-related changes in coordination or precise movement planning (6, 7). It enables the orientation and stability of the body during static and dynamic activities (6, 8).

Awareness of body segments in space is crucial for the body to interact effectively with the environment (6, 9). Proprioception depends on mechanoreceptors located in tendons, ligaments, muscles, and joint capsules (10, 11). Mechanoreceptors transmit information about joint position and movement to the central nervous system (2). Therefore, proprioception is an important factor influencing the quality of life in aging populations. Age-related changes in the peripheral and central nervous systems cause deterioration of proprioceptive mechanisms (2, 10). Two types of tasks are commonly used to assess proprioceptive acuity in clinical and research situations (12). These joint position matching methods involve matching the position of a joint to a reference position, most often without visual input. The first task, “ipsilateral matching” (IPSI), involves determining the reference location and adjusting the arm using memory. This reliance on memory occurs because the reference joint angle, which was demonstrated before the task was performed, is not available during the task.

The second task, “contralateral matching” (CONTRA), takes advantage of the constant presence of the joint’s reference position and that of the other limb. Therefore, memory-based matching in this case does not occur.

Additionally, matching using the opposite limb requires greater interhemispheric communication (or transfer) than in the IPSI task (12). Joint position-matching methods reflect the processing of external sensory feedback and many basic sensorimotor processes (13, 14). Both internal and external feedback are combined in the process of sensorimotor integration involving activation of the somatosensory cortex, primary motor and premotor cortical areas, and subcortical areas (13, 15).

Moreover, cognitive decline represents another significant issue that worsens with age. These declines may be partly dependent on proprioceptive acuity (16). Structural and functional changes at the central level may underlie the decline in proprioceptive performance in older adults (17). Reduced attention, memory, and cognitive resources may diminish proprioceptive acuity in older adults compared to younger adults (17, 18). Age-related declines in cognitive processing ability also contribute to changes in proprioceptive function, particularly in tasks requiring greater cognitive effort (16, 17). Aging leads to a reduction in the dynamic sensitivity of muscle spindles, which affects both position and velocity feedback.

In addition to a reduction in the number of alpha motor neurons, brain areas involved in planning descending motor commands also deteriorate significantly in older people (16, 19).

Improving proprioception in older people is likely possible and may depend on the type of physical training undertaken (18). One activity with a strong association with enhanced proprioceptive abilities is traditional Chinese tai chi (12, 20, 21). Tai chi promotes an increased sense of joint position through slow, deliberate movements and constant awareness of body positioning (20, 21). Similarly, proprioceptive benefits have been reported in older adults who practice activities like golf or creative dancing, which also involve heightened joint position awareness (20, 22). Although little is known about the effects of other forms of physical activity on proprioception in older people (22), juggling shows promise as an activity that can potentially enhance proprioception.

Moreover, data indicate that proprioception contributes greatly to juggling (23). It is an activity that involves throwing and catching balls with both hands simultaneously according to a specific motor pattern (24). Additionally, the neuroplasticity potential of juggling has been confirmed by numerous studies (24–27). There is evidence showing a link between juggling and mental rotation performance and, more broadly, between motor and cognitive performance (28–30). In the juggling cascade, hands toss and catch balls alternately. Combining the limbs into a new phasing relationship requires practice to learn new phasing relationships between limb movements (31). Juggling as a bimanual task requires attention, which can contribute to both proprioception and attention development. Importantly, with greater juggling experience, the direction of focus changes from the hands to the top of the parabolic arc of ball flight (31). An important part of increasing the effectiveness of learning and predicting the trajectory of a ball is focusing one’s attention on the stimuli rather than on the hand (31, 32). Juggling, as an exercise that requires the catching hand to compensate for the throwing hand’s mistakes, appears to perfectly reflect the proprioceptive coordination of two hands. To master juggling, it is crucial to reduce the variability of throws and learn to compensate for errors by producing similar phase relationships of limb movement (31). Notably, performing this physical activity is both appealing and safe for older people (33).

The majority of the studies evaluating the effect of exercise on joint position matching, including the elbow joint, have primarily focused on the effect of acute exercise rather than long-term training (34–37). Additionally, there is limited research on the effect of juggling on brain function and cognitive performance in older adults (26, 38). Therefore, investigating the impact of systematic juggling exercises on elbow joint position adjustment in older women presents a promising area for further exploration.

Given the limited data on juggling interventions in older people, this study aimed to evaluate the effects of juggling on proprioception (through IPSI and CONTRA elbow joint task matching) and assess its impact on attentional abilities in older women, a population known for lower physical activity levels. Moreover, the purpose was to assess the association between attentional abilities and joint position sense. We hypothesized that after physical activity in the form of juggling, there may be a positive change in proprioception in both the IPSI and CONTRA tasks. Moreover, we hypothesized that there may be a significant relationship between attentional abilities and joint position sense, indicating that better attention test results may correlate with better elbow joint position matching results.

## 2 Materials and methods

### 2.1 Participants

A total of 25 right-handed older women responded to announcements about juggling classes, which were made via local radio, television, newspapers, and social media.

Finally, 20 older women were included in the study, which used a repeated measures design based on three conditions (69.95 ± 4.58 years; min-max: 65–76). Five respondents were excluded because they did not meet the inclusion criteria.

The inclusion criteria for participants were as follows: women aged 65 years or older, with no known injuries or pathologies affecting the upper limb, no neurological conditions, and no significant visual impairments. Each participant underwent an interview to confirm that these criteria were met.

Additionally, the handedness of the participants was assessed with the Edinburgh Handedness Inventory – short version (Cronbach’s *α* = 0.93) (39). No dropouts occurred during the study. All participants who started the protocol completed it, and complete measurement data were obtained. The study was conducted in accordance with the Declaration of Helsinki 2013 and approved by the Ethics Committee of Poznan University of Medical Sciences (No. 106/21, date: 04.02.2021). Participants were informed about the procedures before the study commenced. All participants signed a written informed consent form and were informed that they could withdraw from participation at any time. The study was registered retrospectively at ClinicalTrials.gov (NCT06108713). The basic characteristics of the whole group are presented in Table 1.

**Table 1:** Participants characteristics.

Variable	Mean (SD)
Age [years]	69.95 (4.58)
Body mass [kg]	62.88 (8.55)
Body height [cm]	158.65 (5.12)
Body mass index [km/cm^2^]	25.04 (3.52)
Lateralization* [points]	92.50 (12.43)

^*^Assessed by the Edinburgh Handedness Inventory – short version; SD, standard deviation.

### 2.2 Experimental design and intervention

**Figure 1:**
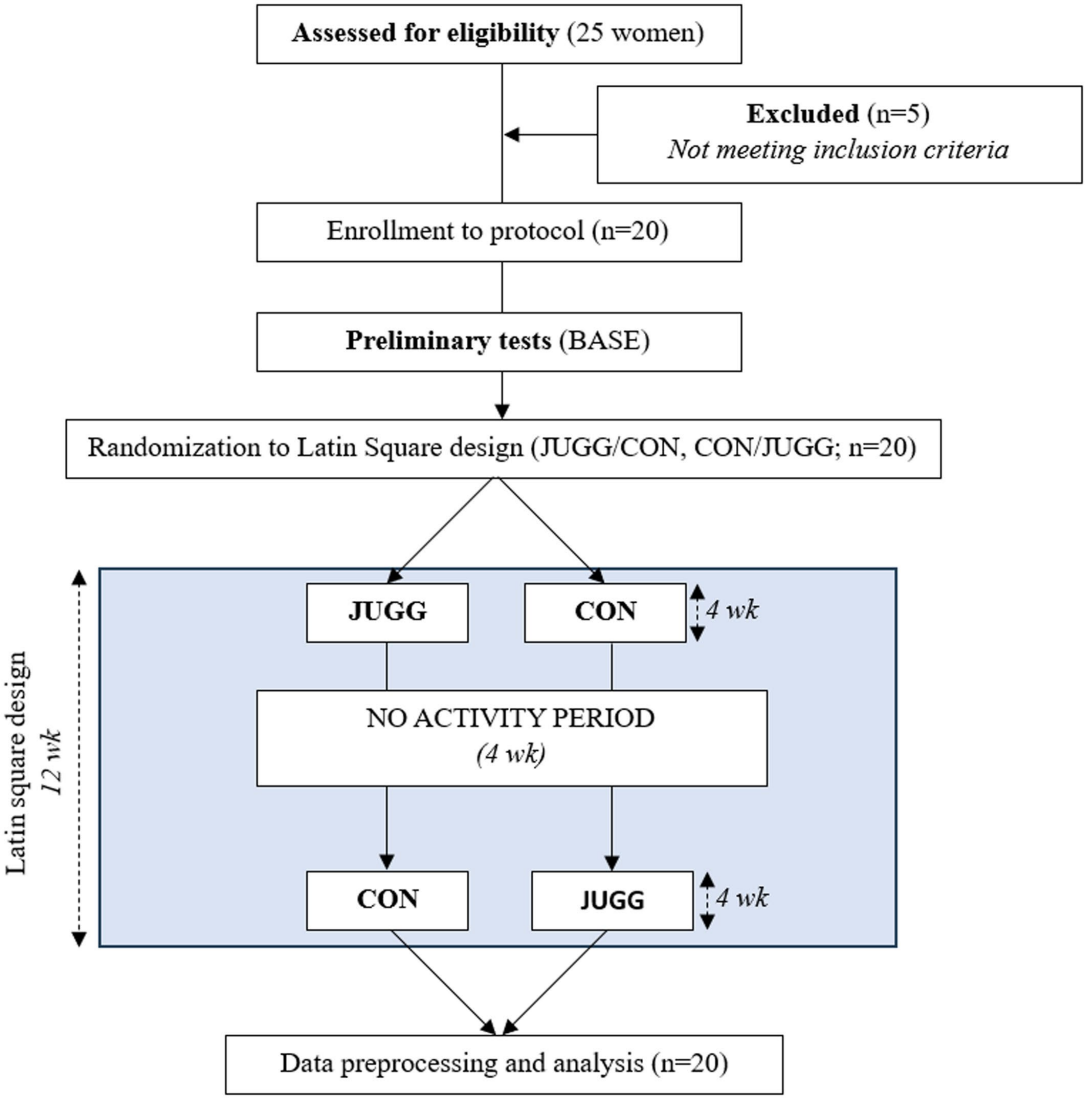
Study design chart; BASE, the series of the first measurements; JUGG, the intervention period with the series of measurements at the end; CON, the control period with the series of measurements at the end; wk, weeks.

The experiment followed a repeated measures design with three measurement time-points (TPs) and an intervention/control condition applied in the assigned sequence order (Figure 1). After the series of the first measurements (baseline, BASE), each participant was randomly assigned to the mentioned different sequence order of intervention/control condition in a crossover manner (each participant experienced both conditions in the assigned sequence), based on the Latin square design (AB/BA). The intervention (A) consisted of four weeks of juggling training, in which participants participated in structured, supervised activities three times a week for 45 min each. The training was a structured juggling activity. The success of the juggling training was defined as the ability to juggle a three-ball cascade. The detailed intervention condition (juggling training) and information on training success were thoroughly described and published elsewhere (33).

The control (B) condition involved four weeks without juggling, and participants were also asked not to undertake any new physical activities. The following two TP measurements (A, B) refer to the series of measurements after each condition. To be precise, the second series of measurements for one sequence order was indeed the third series for the other sequence order, and conversely. To simplify, the series of measurements that occurred after the intervention condition, juggling training, was labeled JUGG, and the series of measurements after the control condition was labeled CON. Additionally, the second sequence was implemented after a four-week break of no activity, during which participants were asked to refrain from juggling or any other new physical activity. A chart of the study design is presented in Figure 1.

#### 2.2.1 Ipsilateral and contralateral position matching

During each measurement meeting for joint position matching, both the IPSI and CONTRA conditions were performed on a special chair that allowed flexion measurement in the elbow joints with an accuracy of 0.1 degrees. Joint flexion was recorded using electrogoniometers, a base unit, and DataLink CP software (Biometrics Ltd.) with a 40 kHz total sampling frequency. This method’s intra-and interrater reliability was described as good (0.76 and 0.86 for flexion, 0.92 and 0.89 for extension, respectively) (41). The chair was individually adjusted to the participants’ body height and limb length, taking into account the shoulders and the axis of the elbow joints. Participants performed eight trials, preceded by three practice trials, with a 30-s break between tasks. For each TP, the results of individual participants in both the IPSI and CONTRA conditions were checked for outliers using the IQR method. Outliers were removed because they may have occurred on a one-time basis if there was less engagement on the task or if there was increasing fatigue. During the whole task, all participants wore a pair of blindfolds that prevented the participants from visually accessing the arm position. While seated upright, both participants’ forearms rested on the movable splints of a bimanual manipulandum, and they were instructed to keep their arms relaxed.

The armrest had a stopper to ensure the same starting position for all the trials (90° flexion of the elbow joint, 80° arm abduction, 10° arm lateral flexion). For both conditions, passive limb displacement was performed in such a way as to achieve a value of 10 degrees of displacement per second. The order of conditions was randomized. The position matching tests allowed us to determine the constant error (CE), absolute error (AE), variability error (VE), and root mean square error (RMSE) for each condition.

The IPSI condition assessed working memory performance during a proprioceptive task. In each IPSI trial, the experimenter moved the splint on the participant’s dominant arm 30 degrees from the starting position toward the body. The experimenter verbally informed the participants when the desired target position was reached. The forearm was then passively moved back to the starting position. Subsequently, the participants actively moved their forearms to the remembered target position. The participants were instructed to stop their movement and verbally indicate when they felt they had reached the reference target. Angular data were recorded immediately after the participant’s command using the potentiometer. They did not receive verbal or visual feedback on the accuracy of their matching performance.

In the CONTRA matching condition, both arms were controlled, and the inversion angle for both arms was kept similar and constant across and within participants. This task aimed to assess the efficiency of interhemispheric proprioceptive information processing during a bimanual proprioceptive task. In each trial, the examiner moved the participants’ non-dominant forearm to a target position of 30 degrees from the starting position toward the body. The examiner verbally informed the participants when the desired target position was reached. The participant’s task was to move their dominant hand to such a position that the elbow flexion angle of the dominant hand matched the elbow flexion angle of the non-dominant hand. The participants held their positions for approximately 3 s to allow for the collection of stable angular data via a potentiometer. They did not receive verbal or visual feedback regarding the precision of their matching performance.

We based the above methods on reports of the general practice of proprioception testing by adjusting joint positions, including that joint position adjustment tasks should be performed at the same magnitude of target amplitudes, should always be adjusted in the same way, and should always be adjusted at the same speed (12).

#### 2.2.2 Reaction time and attention

The results of cognitive abilities were obtained with the Vienna Test System (VTS; SCHUHFRIED GmbH, Austria; Polish distribution - COGNIFIC). Participants performed three different tasks in the following order: simple reaction time test and choice reaction time test (which made it possible to determine simple reaction time and its variability), choice reaction time and its variability, motor time and its variability, decision time and Cognitrone test, which allowed to assess mean time of correct acceptance, mean time of correct rejection, number of correct answers and task duration time. In each measurement meeting, all of the tests were preceded by practice trials.

The simple reaction time test consisted of responding to a visual stimulus displayed on the computer screen (yellow spot). Participants used one finger of the dominant hand to maintain constant contact with the touch panel. After the stimulus was displayed, participants were asked to let go of the touch panel as quickly as possible (reaction time), use the same finger to press the button above the panel, and return to the initial position (motor time).

The choice reaction time test required the same response as the preceding test. Different visual stimuli were presented, but participants had to respond only to a predetermined stimulus. Responding to other stimuli was considered a mistake. The Cronbach’s alpha of the reaction time tests varied between 0.83 and 0.98 for the reaction time results and between 0.84. and 0.95 for the motor time results (SCHUHFRIED GmbH).

The Cognitrone test consisted of finding as quickly as possible among the four figures displayed, the reference figure presented below, and reacting appropriately depending on whether the figure was there (green button under the right hand) or not (red button under the left hand). The level of complexity of the figures presented increased as the test progressed. The test consisted of 60 cases. The Cronbach’s alpha of the Cognitrone test results is 0.95 (SCHUHFRIED GmbH).

### 2.3 Statistical analysis and sample size

The Shapiro–Wilk test was used for data to test the normality of all the data. Differences between three TPs (BASE, JUGG, CON) for each condition separately (IPSI, CONTRA) were checked by repeated measures analysis of variance (ANOVA RM), which included Mauchly’s sphericity analysis. When the sphericity condition was not met, the Greenhouse–Geisser correction was used. Alternatively, Friedman’s analysis of variance (F ANOVA) was used for abnormally distributed data.

After repeated measures analysis, *Post-hoc* tests (Bonferroni or paired Wilcoxon) were used for pairwise comparisons. Differences between conditions were analyzed using paired t-tests for normal distributions or Wilcoxon tests for non-normal distributions. The correlation coefficient was checked by the Spearman correlation coefficient, given the distribution of the variables (nonnormal distribution). The following interpretation of correlation coefficients was applied: less than 0.20 as very weak, 0.20 to 0.40 as weak, 0.40 to 0.60 as medium, 0.60 to 0.80 as strong, and at least 0.8 as very strong.

The results are presented in the tables and figures as means with standard deviations and medians with interquartile ranges (IQR). The effect size for ANOVA RM was determined as partial eta-square (ⴄ_p_^2^; 0.01 – small effect; 0.06 – medium effect; 0.138 – high effect), for F ANOVA, it was determined as Kendall’s coefficient of concordance (W; 0.20 – fair agreement, 0.40 – moderate agreement, 0.60 – substantial agreement, 0.80 – almost perfect agreement), and for pairwise t-tests, it was determined as Cohen’s d (*d*; 0.2 – small effect; 0.5 – medium effect; 0.8 – high effect) or Wilcoxon with bivariate correlation coefficient rank (rc; 0.1 – small effect; 0.3 – moderate effect; 0.5 – large effect). Differences between the means (Md) of paired data are presented with 95% confidence intervals (CI 95%) for all compared variables. A *p*-value of <0.05 was considered to indicate statistical significance. G*Power software (version 3.1.9.6, Germany) was used to calculate the minimum sample size. According to Niespodziński et al. (42), assuming a large effect size, the required sample size at a power of 0.80 is 15 participants. Due to the duration of the study and the possibility of unforeseen situations, we decided to increase this number by 30%. Nevertheless, based on previous studies of elbow joint proprioception in older adults, with sample sizes ranging from 12 to 17 (16, 43–45), the adopted sample size of 20 participants appeared to be sufficient.

## 3 Results

### 3.1 Carryover effect analysis

The carryover effect analysis for the CON condition revealed no significant differences between the sequence order of intervention/control (AB/BA) in the joint position matching tests (for IPSI, *p*-values ranged from 0.473 to 0.970; for CONTRA, *p*-values ranged from 0.307 to 0.678). In terms of cognitive abilities, the carryover effect analysis revealed a significant difference in motor time (*p* = 0.035) between the sequence order of intervention and control. However, no significant differences were found for the other test results (for reaction time tests, *p*-values ranged from 0.175 to 0.950; for Cognitrone test results, *p*-values ranged from 0.266 to 0.921).

### 3.2 Joint position matching

In the IPSI condition, a significant main effect of TPs for CE was observed (*p* = 0.03, ⴄ_p_^2^ = 0.17). The Bonferroni *post-hoc* test for CE in the IPSI condition showed no significant differences between the TPs. The change observed between BASE and the other TPs was very pronounced. The JUGG and CON were characterized by more precise joint position matching. All the TP results were characterized by an overestimation.

In the CONTRA condition, the changes in CE from BASE to JUGG and CON were characterized by deterioration of joint position matching. All TPs were characterized by an underestimation of the position matching the reference position. However, ANOVA RM showed that there was not any statistically significant effect of TPs (*p* = 0.32, ⴄ_p_^2^ = 0.06), which was reflected by the nonsignificant *post hoc* test of differences between TPs.

Data on CE differences across the TP are presented in Table 2. A significant difference between conditions was observed for the CON condition (*p* = 0.01, *d* = 0.64) but not for the BASE (*p* = 0.09; *d* = 0.49) or the JUGG condition (*p* = 0.31; *d* = 0.23). Visualization of changes in CE for each condition and task is shown in Supplementary Figure S1, while data on CE differences between each condition are presented in Table 3. Means with standard deviations of CE are presented in Table 4.

**Table 2:** Mean differences with confidence intervals between the series of measurements (BASE-JUGG; BASE-CON; JUGG-CON).

Conditions	Differences between the series of measurements		
	BASE-JUGG Md [95%CI] *p*-value	BASE-CON Md [95%CI] *p*-value	JUGG-CON Md [95%CI] *p*-value
CE			
IPSI	2.08 [−0.13;4.30] 0.072	2.14 [−0.07;4.36] 0.061	0.61 [−2.16;2.28] 1.000
CONTRA	0.71 [−2.70;4.12] 1.000	2.04 [−1.36;5.45] 0.424	1.33 [−2.07;4.74] 0.999
AE			
IPSI	1.27 [0.26;2.28] 0.010*	1.32 [0.31;2.33] 0.007*	0.05 [−0.96;1.06] 1.000
CONTRA	−0.54 [−2.65;1.56] 1.000	0.69 [−1.42;2.80] 1.000	1.23 [−0.87;3.34] 0.454
VE			
IPSI	5.43 [−0.59;11.44] 0.089	4.68 [−1.33;10.70] 0.176	−0.74 [−6.76;5.27] 1.000
CONRA	5.96 [−2.83;14.75] 0.293	4.28 [−4.51;13.08] 0.690	−1.68 [−10.47;7.11] 1.000
RMSE			
IPSI	1.36 [0.28;2.44] 0.010*	1.30 [0.22;2.39] 0.014*	−0.57 [−1.14;1.03] 1.000
CONTRA	−0.28 [−2.45;1.89] 1.000	0.81 [−1.36;2.98] 1.000	1.09 [−1.08;3.26] 0.665

^*^statistically significant; IPSI, the ipsilateral condition of joint position matching; CONTRA, the contralateral condition of joint position matching; BASE, the first series of measurements; JUGG - the series of measurements taken after the intervention period; CON, the series of measurements taken after a period without implementing any intervention; Md, mean difference; 95%CI, 95% confidence interval of mean difference; CE, constant error of joint position matching assessment; AE, absolute error of joint position matching assessment; VE, variable error of joint position matching assessment; RMSE, root mean square error of joint position matching assessment.

**Table 3:** Mean differences with confidence intervals between joint position matching conditions (IPSI-CONTRA).

Conditions	BASE Md [95%CI] *p*-value	JUGG Md [95%CI] *p*-value	CON Md [95%CI] *p*-value
CE	2.83 [−0.44;6.11] 0.086	1.46 [−1.48;4.40] 0.312	2.73 [0.72;4.74] 0.010*
AE	−0.83 [−2.31;0.64] 0.252	−2.65 [−4.46;−0.83] 0.007*	−1.46 [−2.76;−0.17] 0.029*
VE	2.87 [−10.86;5.12] 0.461	−2.34 [−7.56;2.89] 0.361	−3.27 [−9.99;3.44] 0.302
RMSE	−1.03 [−2.51;0.45] 0.191	−2.67 [−4.51;−0.84] 0.007*	−1.52 [−2.93;−0.12] 0.035*

^*^Statistically significant; IPSI, the ipsilateral condition of joint position matching; CONTRA, the contralateral condition of joint position matching; BASE, the first series of measurements; JUGG, the series of measurements taken after the intervention period; CON, the series of measurements, taken after a period without implementing any intervention; Md, mean difference; 95%CI, 95% confidence interval of mean difference; CE, constant error of joint position matching assessment; AE, absolute error of joint position matching assessment; VE, variable error of joint position matching assessment; RMSE, root mean square error of joint position matching assessment.

**Table 4:** IPSI and CONTRA means with standard deviations obtained in three different TPs.

Variables	BASE Mean ± SD	JUGG Mean ± SD	CON Mean ± SD	BASE-JUGG %	BASE-CON %	f/*X^2^* *p*-value [ES]
IPSI_CE [^o^]	2.73 ± 2.92	0.64 ± 2.93	0.58 ± 3.12	↓ 124.04	↓ 129.91	3.80 0.03* [0.17]
CONTRA_CE [^o^]	−0.10 ± 5.63	−0.81 ± 6.75	−2.15 ± 4.38	↓ 156.04	↓ 182.22	1.16 0.32 [0.06]
IPSI_AE [^o^]	4.52 ± 1.12^J,C^	3.25 ± 1.12^B^	3.20 ± 1.53^B^	↓ 32.69	↓ 34.20	*11.70* <0.01* [0.29]
CONTRA_AE [^o^]	5.36 ± 2.77	5.90 ± 3.66	4.67 ± 2.08	↑ 9.59	↓ 13.76	*0.30* *0.86 [0.01]*
IPSI_VE [^o^]	13.51 ± 9.54	8.08 ± 4.95	8.83 ± 7.15	↓ 50.30	↓ 48.57	*1.20* 0.55 [0.03]
CONTRA_VE [^o^]	16.38 ± 14.78	10.42 ± 9.19	12.10 ± 12.50	↓ 47.48	↓ 30.06	*4.30* 0.12 [0.11]
IPSI_RMSE [^o^]	5.11 ± 1.11^J,C^	3.75 ± 1.27^B^	3.81 ± 1.71^B^	↓ 30.70	↓ 29.15	6.33 <0.01* [0.25]
CONTRA_RMSE [^o^]	6.14 ± 2.81	6.42 ± 3.57	5.33 ± 2.22	↑ 4.56	↓ 14.12	*0.70* *0.70 [0.02]*

*Statistically significant; ^B^result significantly different from baseline time-point; ^J^result significantly different from juggling time-point; ^C^result significantly different from control time-point; TPs, time-points; BASE, baseline time-point; JUGG, juggling time-point; CON, control time-point; IPSI, the ipsilateral condition; CONTRA, the contralateral condition; CE, constant error; AE, absolute error; VE, variable error; RMSE, root mean square error f/*X^2^*, f statistic or chi-square statistic (if in italics); ES, effect size: ⴄ^2^ (f statistic) or W (chi-square statistic).

A significant difference in AE was observed in the IPSI condition (*p* < 0.01, W = 0.29), with worse AE scores observed in the BASE condition compared to the other TPs, indicating a disadvantage for BASE. Wilcoxon pairwise comparison revealed significant differences between BASE, as well as between JUGG and between BASE and CON, but not between JUGG and CON.

For the CONTRA condition, no statistical significance was observed in the F ANOVA analysis (*p* = 0.86, W = 0.01), which was corroborated by Wilcoxon pairwise comparisons. The percentage difference indicated a minor advantage for CON and a slight disadvantage for JUGG compared to BASE. Data on AE differences across TPs are presented in Table 2.

Between the conditions, a statistical difference was observed for JUGG (*p* < 0.01, *d* = 0.68) and CON (*p* < 0.05, *d* = 0.44) but not for BASE (*p* = 0.43, rc = 0.23). All AE data are presented in Supplementary Figure S2, and data on AE differences between all conditions are presented in Table 3. Means with standard deviations for AE are presented in Table 4.

F ANOVA showed no statistically significant main effect of TPs for the IPSI condition in the VE (*p* = 0.55, ⴄ_p_^2^ = 0.03). The lack of significant changes was further confirmed by the results of Wilcoxon pairwise comparisons. However, the overall percentage change between the BASE and the other conditions showed noticeable improvement in later TPs.

In the CONTRA condition, no statistical significance was observed (*p* = 0.12, ⴄ_p_^2^ = 0.11). Compared to BASE, an approximation to the reference value was observed for JUGG and CON. Data on VE differences between each TP are presented in Table 2.

No statistically significant differences were detected across the TPs (BASE: *p* = 0.79, rc = 0.06; JUGG: *p* = 0.71, rc = 0.08; CON: *p* = 0.43, rc = 0.17). All results from the VE analysis are presented in Supplementary Figure S3, with data on VE differences between conditions presented in Table 3. Means with standard deviations for AE are presented in Table 3.

ANOVA RM showed a statistically significant difference in RMSE for the IPSI condition (*p* < 0.01, ⴄ_p_^2^ = 0.25). The results recorded in both the JUGG and CON were noticeably closer to the reference values than those recorded in the BASE group. Bonferroni *post hoc* pairwise comparisons detected differences between BASE and JUGG and between BASE and CON. Differences between JUGG and CON were not statistically significant.

In the CONTRA condition for the RMSE, F ANOVA was used. No statistical significance was detected between TPs (*p* = 0.70, W = 0.02). However, the difference between the TPs indicated that BASE performed worse. Data of RSME differences between each TP are presented in Table 2.

Differences between conditions were observed in JUGG (*p* < 0.01, *d* = 0.68) and CON (*p* = 0.04, *d* = 0.51) but not in BASE (*p* = 0.19, rc = 0.29). All the RMSE data are presented in Supplementary Figure S4, data of RMSE differences between each condition are presented in Table 3, and means with standard deviations of RMSE are presented in Table 4.

### 3.3 Cognitive abilities

The only statistically significant change was observed for correct reactions in the Cognitrone test_._ All the cognitive test data are presented in Table 5.

**Table 5:** Results of cognitive tests in three different TPs.

Variables	BASE Mean ± SD *Median ± IQR*	JUGG Mean ± SD *Median ± IQR*	CON Mean ± SD *Median ± IQR*	*p*-value [ES]
Simple reaction time [ms]	302.10 ± 48.65 *294.00 ± 57.00*	296.55 ± 41.33 *292.00 ± 53.00*	293.60 ± 43.32 *287.00 ± 70.00*	0.63 [0.02]
Variability of simple reaction time [ms]	41.10 ± 17.30 *37.00 ± 16.00*	40.55 ± 39.40 *38.50 ± 14.00*	39.40 ± 14.64 *34.50 ± 22.50*	*0.85* *[0.01]*
Choice reaction time [ms]	491.60 ± 56.66 *482.50 ± 67.50*	472.70 ± 50.10 *473.50 ± 78.00*	478.80 ± 52.96 *472.50 ± 50.00*	*0.45* *[0.04]*
Variability of choice reaction time [ms]	72.65 ± 16.66 *68.00 ± 14.50*	67.60 ± 19.69 *65.50 ± 33.00*	72.80 ± 21.07 *70.00 ± 27.00*	0.54 [0.03]
Motor time [ms]	263.38 ± 57.05 *254.75 ± 88.75*	265.55 ± 68.96 *247.50 ± 106.75*	260.28 ± 75.63 *251.25 ± 94.25*	0.87 [0.01]
Variability of motor time [ms]	37.15 ± 7.95 *38.00 ± 12.00*	35.40 ± 11.71 *35.25 ± 13.50*	35.45 ± 14.15 *31.75 ± 15.00*	*0.54* *[0.03]*
Decision time [ms]	189.50 ± 48.15 *176.00 ± 70.00*	176.15 ± 49.45 *166.50 ± 76.50*	185.20 ± 33.65 *190.50 ± 54.00*	*0.82* *[0.01]*
Correct rejections in the Cognitrone test [s]	3.19 ± 0.62 *3.22 ± 1.10*	3.11 ± 0.77 *3.07 ± 1.18*	3.11 ± 0.76 *3.02 ± 0.86*	0.78 [0.01]
Correct acceptances in the Cognitrone test [s]	2.58 ± 0.67 *2.39 ± 0.68*	2.50 ± 0.61 *2.41 ± 0.83*	2.52 ± 0.61 *2.41 ± 0.64*	*0.86* *[0.01]*
Correct reactions in the Cognitrone test [points]	53.75 ± 2.92 *54.00 ± 4.00*	55.65 ± 3.76 *57.00 ± 6.00*	55.90 ± 4.28 *56.50 ± 5.00*	0.01* [0.21]
Duration time of the Cognitrone test [s]	182.45 ± 47.42 *174.00 ± 46.00*	171.90 ± 35.24 *171.50 ± 56.50*	171.95 ± 34.83 *172.50 ± 34.50*	*0.21* *[0.08]*

^*^Statistically significant; BASE, baseline time-point; JUGG, juggling time-point; CON, control time-point; ES, effect size: ⴄ^2^ (f statistic) or W (if in italics; chi square statistic).

### 3.4 Correlation coefficient

Spearman’s Rho analysis revealed statistically significant correlations for the CONTRA condition: a weak negative correlation between CE and simple reaction time, a weak positive correlation between CE and variability in choice reaction time, and a medium positive correlation between CE and decision time. Additionally, variability in choice reaction time was weakly and negatively correlated with AE and RMSE in the CONTRA condition. In the IPSI condition, a weak negative correlation was found between handedness and both AE and RMSE. No other variables were significantly associated with any type of error in either the IPSI or CONTRA conditions. The results of the correlation analysis between cognitive variables and joint position matching are presented in Table 6.

**Table 6:** Correlation coefficient between join position matching conditions and cognitive abilities.

Variables	IPSI Spearman rho (*p*-value)				CONTRA Spearman rho (*p*-value)			
	CE	AE	VE	RMSE	CE	AE	VE	RMSE
Simple reaction time	−0.06 (0.62)	−0.02 (0.86)	0.11 (0.38)	−0.02 (0.91)	−0.36* (<0.01)	0.20 (0.13)	0.05 (0.68)	0.19 (0.14)
Variability of simple reaction time	−0.03 (0.83)	0.01 (0.94)	0.19 (0.14)	−0.01 (0.91)	−0.24 (0.07)	0.17 (0.21)	−0.03 (0.79)	0.16 (0.21)
Choice reaction time	−0.01 (0.91)	0.07 (0.58)	−0.01 (0.93)	0.05 (0.73)	0.08 (0.52)	0.02 (0.86)	0.04 (0.75)	0.01 (0.93)
Variability of choice reaction time	0.24 (0.07)	0.17 (0.19)	−0.05 (0.69)	0.13 (0.34)	0.39* (<0.01)	−0.27* (0.04)	0.00 (1.00)	−0.26* (0.05)
Motor time	−0.18 (0.18)	0.02 (0.85)	0.02 (0.88)	0.03 (0.83)	−0.13 (0.34)	0.09 (0.48)	0.09 (0.50)	0.09 (0.48)
Variability of motor time	0.01 (0.94)	0.13 (0.32)	0.06 (0.66)	0.11 (0.42)	0.09 (0.51)	0.01 (0.92)	0.19 (0.14)	0.03 (0.82)
Decision time	0.07 (0.60)	0.13 (0.31)	−0.02 (0.88)	0.09 (0.49)	0.42* (<0.01)	−0.19 (0.14)	−0.06 (0.63)	−0.22 (0.09)
Correct rejections in the Cognitrone test	−0.05 (0.73)	−0.06 (0.73)	−0.10 (0.47)	−0.07 (0.61)	−0.09 (0.48)	0.15 (0.27)	0.03 (0.80)	0.13 (0.32)
Correct acceptances in the Cognitrone test	−0.05 (0.72)	−0.07 (0.58)	0.03 (0.85)	−0.08 (0.55)	−0.19 (0.15)	0.18 (0.16)	−0.04 (0.79)	0.17 (0.19)
Correct answers in the Cognitrone test	−0.20 (0.13)	−0.14 (0.29)	−0.07 (0.58)	−0.13 (0.33)	−0.21 (0.11)	0.04 (0.77)	−0.25 (0.06)	−0.01 (0.95)
Time duration of the Cognitrone test	−0.02 (0.85)	0.01 (0.95)	−0.02 (0.90)	0.00 (1.00)	−0.11 (0.40)	0.09 (0.49)	−0.14 (0.30)	0.08 (0.55)
Handedness	0.09 (0.51)	−0.27* (0.04)	−0.24 (0.07)	−0.30* (0.02)	0.08 (0.56)	0.20 (0.14)	0.16 (0.23)	0.20 (0.13)

^*^Statistically significant; IPSI, the ipsilateral joint position matching task; CONTRA, the contralateral joint position matching task; CE, constant error; AE, absolute error; VE, variable error; RMSE, root mean square error.

## 4 Discussion

### 4.1 Joint position matching

The main purpose of this study was to determine the effect of additional juggling exercises on joint position-matching tasks and cognitive abilities in healthy, physically active women older than 65 years. The assessment was performed in BASE, JUGG, and CON. The results showed minor improvements in joint position-matching accuracy in the JUGG group.

Similar changes were also observed in the CON group, especially for CE and VE in all conditions, as well as for AE and RMSE in the IPSI condition. We hypothesized that physical activity in the form of juggling would have a positive effect on the accuracy of elbow position matching among older women. Thus, we found that the juggling training intervention did not improve the accuracy of elbow joint position matching among the older women studied.

A systematic review (40) of studies on improving proprioception (including balance) showed that positive effects could be observed after just 3 weeks of intervention. However, in studies considering the effects of whole-body exercises on proprioception in older adults, the most common duration of intervention varied from 6 weeks to 12 months.

The lack of significant effects of juggling on elbow position matching in our study could be due to the intervention being too short. The secondary aim of this study was to evaluate the correlations between attention and reaction time variables and type of error in two different joint position matching tasks. A significant correlation was observed mostly for CE in the CONTRA condition. These were weak or medium correlations.

Few studies have assessed the effect of specific activities on the proprioception of the upper limbs among older women. In particular, research on juggling, which may have a promising impact on neuroplasticity, is lacking (24). As such, this discussion is based on the limited available literature.

Age-related changes in proprioceptive abilities are well-documented (12, 16, 18, 52). Although the potential for physical activity to improve proprioception is increasingly recognized (18, 20–22, 52), there remains a need to explore new types of physical activities that can influence these abilities. Studies (16, 52) have shown that active older adults tend to have better positional accuracy scores than their more sedentary counterparts.

The degree of AE results in studies conducted by Adamo D.E. et al. (16, 52) is comparable to the scores of our participants, especially at the BASE. However, in later TPs for the IPSI condition, all error variables showed a noticeable improvement for both the JUGG and CON groups compared to the BASE, with statistically significant improvements observed for AE, CE, and RMSE. A similar trend of difference was also observed in VE, although the differences were not statistically significant.

This finding suggests that, at least in older women, familiarity with a task may enhance performance in proprioceptive tasks over time. Given the lack of differences between the JUGG and CON groups, it is difficult to determine whether the intervention itself contributed to this effect. One possible explanation is the “familiarity effect,” as research suggests older adults are more inclined to engage in activities with which they are familiar (46).

In the CONTRA condition, no statistically significant changes were observed for any type of error. However, a trend emerged, indicating a deterioration in performance for both JUGG and CON compared to BASE. Interestingly, despite this decline, the JUGG group remained close to the reference values.

Compared to BASE, JUGG showed deterioration in each error type, while in CON, AE, VE, and RMSE improved noticeably relative to BASE. These differences, particularly the decline in accuracy for JUGG, are intriguing. Given the documented brain changes following juggling training—such as increased gray matter volume in the visuomotor complex (24–26, 47)—the observed difference may be related to neuroplastic changes that occur in the brain after bimanual tasks (48–50). Another possibility is that participants had not previously engaged their upper limbs to the same extent as during the juggling intervention, leading to changes in limb activation (53). However, since these differences were not statistically significant, further research is needed to explore changes in interhemispheric communication during bimanual task improvement.

Of the two joint position matching conditions used, previous scientific data (12) indicated that the smallest AE, relative to the reference value, should be characteristic of the IPSI condition, which involves memory requirements. In contrast, higher AE values are usually observed in the CONTRA condition, during which memory is no longer involved. However, this condition requires more interhemispheric interaction during task performance (12). In our research, these speculations were confirmed. We observed significant differences between conditions in both JUGG and CON for AE and RMSE. The BASE was characterized by no significant differences between conditions for all types of error but better accuracy between conditions. Thus, it can be speculated that the interhemispheric interaction that occurs during the CONTRA condition in older women affects both the accuracy of item matching and the repeatability of matching attempts.

### 4.2 Cognitive abilities

In the Vienna Test System, the only significant change was observed in the number of correct answers on the Cognitrone test. However, no significant differences were found between the JUGG and CON groups in terms of correct answers. The results for other cognitive abilities did not differ significantly across all TPs.

However, most variables showed better (though not statistically significant) results across the TPs following the familiarization phase. This suggests that, similar to joint position matching, familiarity with the tasks prior to the main research sessions is essential for obtaining reliable results in cognitive testing. This trend highlights the importance of conducting familiarization sessions before the primary evaluations to ensure more accurate and consistent outcomes.

In summary, the juggling training intervention did not have any measurable effect on the cognitive abilities of older women studied in the chosen cognitive tests. Previous studies (54–56) have shown that physical activity can significantly improve cognitive abilities, especially attention (55, 56). Unfortunately, our results did not support the idea that juggling produces similar cognitive benefits in older adults. However, research on healthy older adults (47–50) has demonstrated that the effect size of cognitive changes following physical activity interventions varies, ranging from non-significant (47, 48) to 0.48 and higher (56–58). It is likely that older, physically active women may be a population in which changes in cognitive abilities are more difficult to discern.

### 4.3 Correlation results

Correlation analysis revealed that in the IPSI condition, a higher degree of right-handedness was associated with greater accuracy in position matching with the right hand. However, attentional abilities and reaction time were not related to accuracy in the joint position-matching task in this condition. This could mean that for people who use their left hand less frequently in daily activities, the right hand may exhibit significantly better accuracy in detecting position. Moreover, it appears that the participant’s reaction speed or fluctuations in attentional abilities do not have a substantial impact on elbow joint position matching in the IPSI condition.

In contrast, the CONTRA condition showed no relationship with handedness, which confirms that this task requires communication between both hemispheres (12). Correlation analysis showed that participants who had slower reaction times tended to underestimate position matching in the CONTRA condition. An inverse relationship was observed for variability in choice reaction time and decision time: greater variability in response to stimuli requiring decisions and longer decision-making times were associated with respondents’ tendency to overestimate the elbow position matching task in the CONTRA condition.

Interestingly, variability in choice reaction time was negatively correlated with errors in item matching accuracy (AE and RMSE), suggesting that less variability was associated with better joint position-matching performance. This discrepancy may be due to similar decision times across trials requiring choices between several stimuli. Thus, longer decision-making times may lead to overestimations in the joint position-matching tasks. However, greater variability in decision response times was linked to fewer errors in joint position matching in the CONTRA condition. These findings suggest that improvements in the consistency of choice reaction time and decision time are associated with better position-matching accuracy in the CONTRA condition. Therefore, it may be worth considering adjustments for decision time when evaluating elbow joint position matching in the CONTRA condition, as it could significantly influence the results.

No significant correlations were found between attention tasks measured by the Cognitrone test and performance in the CONTRA condition, indicating that attention may not play a critical role in elbow position adjustment tasks in either the IPSI or CONTRA condition. Further research is needed to explore the role of attention in joint position-matching tasks more comprehensively.

### 4.4 Limitations

Notably, our research has several limitations. We assessed the effects of juggling only in a group of physically active women over 65 years of age. The participants were those who voluntarily responded to announcements about the classes, making them unlikely to represent the broader population of older women. Instead, they are probably more representative of individuals motivated to engage in new activities.

Additionally, changes in cognitive functioning and proprioceptive abilities can occur at different rates within a wide age range (65–76 years). Including both genders in a comparative analysis would likely have enhanced the quality and generalizability of the results. Notably, although the sample size was consistent with estimation requirements and larger than many studies on elbow joint position matching, it may still have been insufficient to detect clear differences for variables with such subtle effects.

The chair used to measure elbow joint position was a custom-made instrument that may have deviated from common standards. However, this design allowed for precise adjustments to accommodate participants’ body heights and individual limb segment lengths. Despite using reliable tools to measure elbow flexion, the custom-made chair may have slightly reduced the reliability of these tools. Moreover, the maximal force in the upper extremities—used to condition muscle spindles before the joint position matching test—was only measured during the BASE phase, potentially changing throughout the study.

Due to the length of the measurement sessions, we chose to assess joint position matching only with the dominant hand, which unfortunately limits the scope of the findings compared to examining both limbs under the same conditions.

### 4.5 Strengths

A key strength of our study was the design, which included familiarizing the participants with proprioception and cognitive ability tests during separate TPs. Notably, participants’ performance in both the IPSI and CONTRA conditions often deviated from the reference values at the BASE, while better results were observed in the JUGG and CON conditions. To obtain reliable results in proprioception testing, particularly with the elbow joint position adjustment method, it may be beneficial to conduct the familiarization session on a different day from the main test, especially with older adults.

An additional advantage of our study was the inclusion of average differences between TPs and the percentage range of change across conditions, providing a comprehensive view of the changes within study group. To the best of our knowledge, this is the first time that outliers for individual trials were rejected based on an internal analysis of the participants’ attempts at a given TP, rather than discarding all results from a participant due to a single outlier in the group.

While this approach may have contributed to the normalization of the study group’s results, it also risks excluding individuals who could represent an interesting segment of the population, thus limiting insights. In addition, correlating the results of attention and reaction time tests with joint position matching performance provided a more detailed understanding of the relationship between cognitive and proprioceptive abilities, an area that warrants further investigation in further research using joint position-matching tasks.

## 5 Conclusion

The present study did not confirm a significant effect of juggling on joint position matching in either the IPSI or CONTRA conditions in healthy, physically active older women. No differences were found between the JUGG and CON groups in terms of reaction time, attention, or joint position-matching accuracy. However, some fluctuations were observed, suggesting a possible influence of the bimanual task on participants. A noticeable improvement in variable error (VE) was seen following juggling, but the lack of statistical significance prevents drawing firm conclusions for the wider population.

Additionally, relationships between variables pointed to a potential role of decision speed and the stability of choice reaction time in achieving more accurate position representation. However, no evidence was found regarding a correlation between attention and joint position matching in the IPSI condition. These relationships warrant further investigation in future research.

## Data Availability

The raw data supporting the conclusions of this article will be made available by the authors, without undue reservation.
