# Supplementary material for: The effect of juggling on the proprioceptive and attentional abilities among older women
Source: Front Public Health. 2024 Oct 2;12:1386981. doi: 10.3389/fpubh.2024.1386981 (PMC11479956; doi:10.3389/fpubh.2024.1386981)
Supplement: Supplementary file 1 [file Image_1.PDF]

Supplementary Material

The effect of juggling on the proprioceptive and attentional abilities among older women

Jakub Malik*, Natalia Główka, Wojciech Jelonek and Janusz Maciaszek

*** Correspondence:** Corresponding Author: malik@awf.poznan.pl

1 Supplementary Figures and Tables

1.1 Supplementary Figures

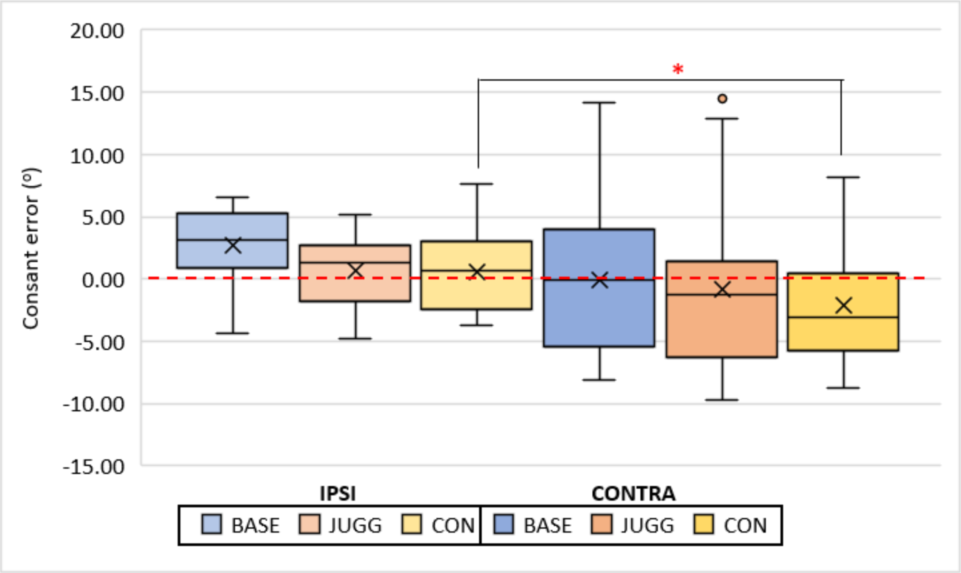

Supplementary Figure 1. Box plots with outliers: constant error of joint position matching test.
* - statistically significant difference; IPSI – the ipsilateral condition of joint position matching; CONTRA – the contralateral condition of joint position matching; BASE – the first series of measurements; JUGG – the series of measurements, taken after the intervention period; CON – the series of measurements, taken after a period without implementing any intervention;

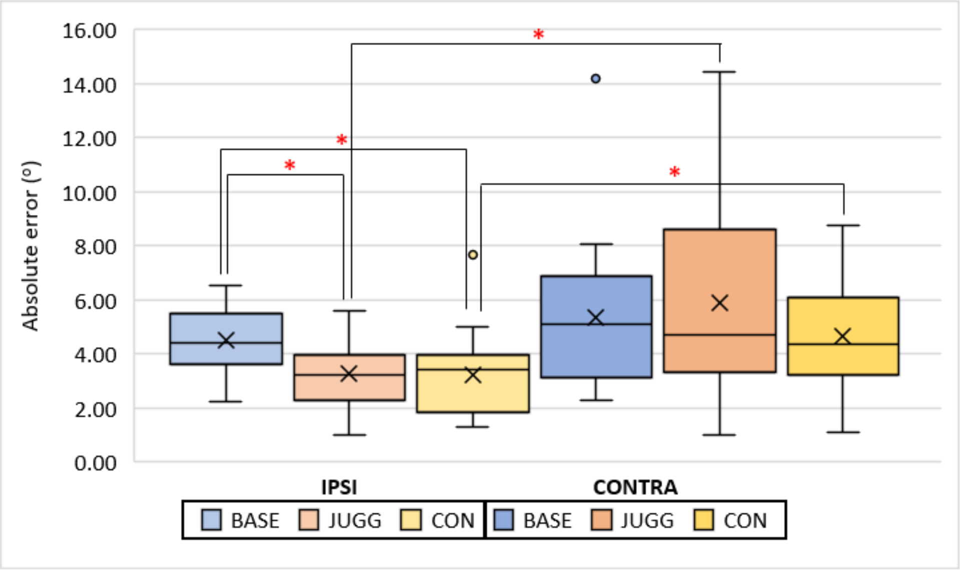

Supplementary Figure 2. Box plots with outliers: absolute error of the joint position matching test. * - statistically significant difference; IPSI – the ipsilateral condition of joint position matching; CONTRA – the contralateral condition of joint position matching; BASE – the first series of measurements; JUGG - the series of measurements, taken after the intervention period; CON – the series of measurements, taken after a period without implementing any intervention;

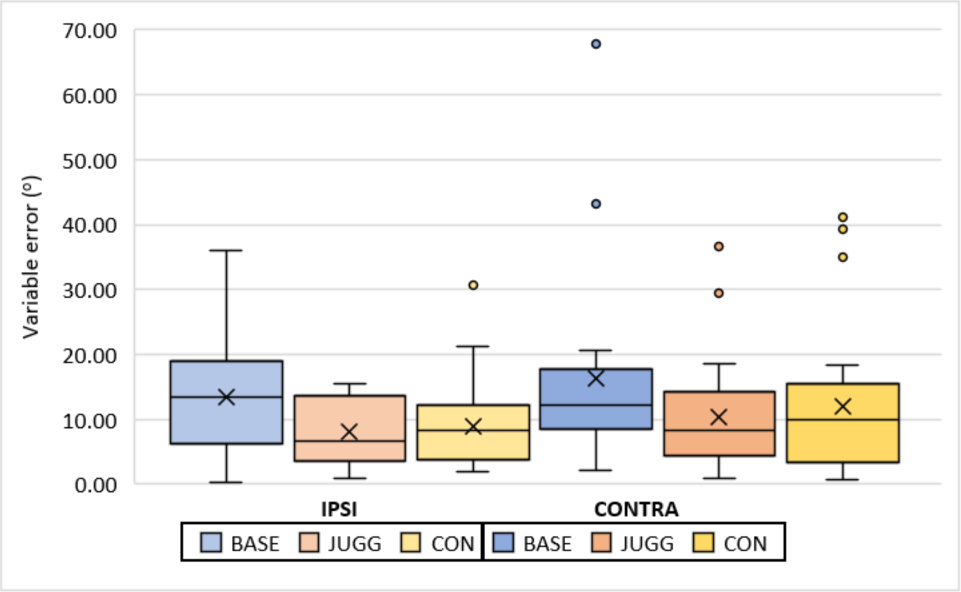

Supplementary Figure 3. Box-plots with outliers: variable error in the joint position matching test. IPSI – the ipsilateral condition of joint position matching; CONTRA – the contralateral condition of joint position matching; BASE – the first series of measurements; JUGG - the series of measurements, taken after the intervention period; CON – the series of measurements, taken after a period without implementing any intervention;

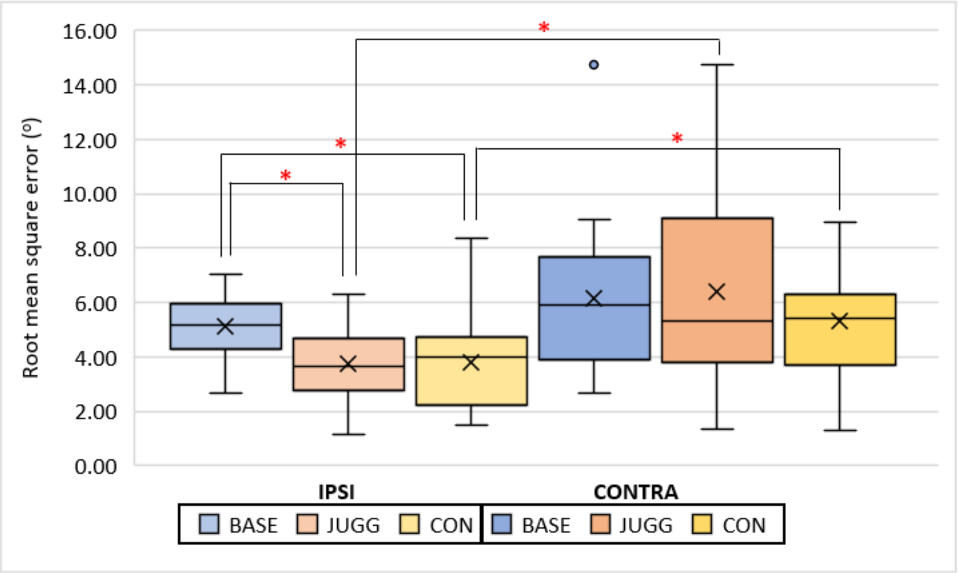

Supplementary Figure 4. Box plots with outliers: root mean square error of the joint position matching test.

* - statistically significant difference; IPSI – the ipsilateral condition of joint position matching; CONTRA – the contralateral condition of joint position matching; BASE – the first series of measurements; JUGG - the series of measurements, taken after the intervention period; CON – the series of measurements, taken after a period without implementing any intervention;
